# The Questions on Everyone’s Mind: What is and Why Do We Need Preventive Cardiology?

**DOI:** 10.14797/mdcvj.698

**Published:** 2021-09-24

**Authors:** Tahir Mahmood, Michael D. Shapiro

**Affiliations:** 1 Oregon Health & Science University, Portland, Oregon, US; 2 Wake Forest University School of Medicine, Winston-Salem, North Carolina, US

**Keywords:** atherosclerotic cardiovascular disease, diabetes, dyslipidemia, hypertension, medical subspecialty, obesity, preventive cardiology, risk assessment

## Abstract

Despite being largely preventable, atherosclerotic cardiovascular disease (ASCVD) continues to be the leading source of morbidity and mortality worldwide. While the past few decades have seen a substantial reduction in ASCVD mortality, much of this is due to significant improvements in treatment of already manifest disease, with its attendant morbidity and impact on quality of life. Moreover, evidence now suggests that ASCVD mortality in the United States has hit a nadir and is likely to start increasing again. It is now time to shift our attention from intervention to prevention. In this review, we explore the tremendous opportunity to define and implement the discipline of preventive cardiology.

## Introduction

Atherosclerotic cardiovascular disease (ASCVD) remains the leading cause of both morbidity and mortality worldwide. In 2019 alone, there were roughly 523.2 million cases and 18.6 million deaths attributed to cardiovascular disease globally.^1^ The financial burden of CVD is equally enormous, with direct costs exceeding $200 billion annually in the United States alone.^1^ While there is certain age-related risk, the fact that ASCVD is largely preventable makes these numbers rather astonishing to consider.

The overarching strategies for primary ASCVD prevention are seemingly intuitive—maintaining a healthy diet and normal weight, engaging in regular exercise, tobacco cessation, and regular monitoring of blood pressure, cholesterol, and blood sugar.^2^ However, actual implementation and monitoring of these preventive strategies in medical practice has been oversimplified and suboptimal.^3^ Prime examples include the vast underdiagnosis of obesity and familial hypercholesterolemia in primary care throughout the world.^4,5,6,7^ Our limited effectiveness in preventing ASCVD coupled by its immense burden have driven recent efforts to establish a dedicated preventive cardiology subspecialty.^8^ In this review, we contextualize the need for preventive cardiology and highlight emerging ideas within the developing field.

## Concerning Trends in Cardiovascular Disease Mortality

ASCVD mortality in the United States has substantially reduced over the past four decades,^1^ but this comes with two notable caveats. First, about half of these reductions have been attributed to improvements in the care and treatment of already established ASCVD.^9^ We experienced significant breakthroughs in systems of care, including advances in surgical and interventional techniques, decreased door to balloon time in ST-elevation myocardial infarctions, development of cardiac care units, and use of novel medications for secondary prevention. Nonetheless, these innovations allow patients to live longer with significant morbidity, and many can experience remarkable reductions in quality of life. For example, a recent study found that older adults with CVD have higher rates of rapid functional decline compared to those without (23.8% vs 16.2%).^10^ Of course, a more prudent strategy would be to focus more of our energies on preventing ASCVD in the first place.

The second and perhaps more alarming caveat is that reductions in ASCVD mortality appear to be hitting a nadir in the United States. Evidence suggests that the rate of ASCVD mortality decline has significantly slowed in many high-income countries. In fact, ASCVD mortality appears to actually be rising in US adults aged 35 to 74 years.^11^ The most obvious reason for this is the high and increasing levels of obesity and diabetes, which remain growing universal epidemics.^12^ Smoking prevalence is also low enough in the United States that further declines are having a relatively smaller impact on mortality. Lastly, it is important to keep in mind that persistent inequities exist in ASCVD risk, many of which are grounded in socioeconomic disparities.^13^ Together, these factors pose significant challenges to public policymakers to promote better cardiovascular health, and they certainly play a role in the stagnating ASCVD mortality trends in the United States.

It would be remiss to ignore the intersection between ASCVD and the ongoing COVID-19 pandemic.^14^ A recent large meta-analysis demonstrated that ASCVD and its risk factors are associated with increased mortality from COVID-19 infection across all age groups.^15^ Accordingly, the Centers for Disease Control and Prevention currently lists heart conditions (including coronary artery disease), obesity, smoking, and type 2 diabetes mellitus among conditions that increase risk for severe illness from COVID-19.^16^ The COVID-19 pandemic not only emphasizes the unfortunate pervasiveness of ASCVD comorbidity throughout the medical system but also underscores the need for a more comprehensive and dedicated approach to ASCVD prevention overall, especially to help control future public health crises.^17^

## Historical Context of Preventive Cardiology

Paradigms surrounding ASCVD prevention have undergone multiple shifts within the past century. The early stages of ASCVD prevention can be traced back to 1948 with the initiation of the Framingham Heart Study.^18^ It was within this landmark epidemiological study that traditional modifiable risk factors associated with ASCVD—including hypercholesterolemia, hypertension, diabetes, and smoking—were first identified and popularized.^19^ Ultimately, identification of these risk factors helped to establish the well-known Framingham Risk Score^20^ and have formed the basis of modern 10-year quantitative risk assessment in primary prevention.^21^

Ensuing clinical trials evaluated lifestyle modifications and medications to control these risk factors, and early evidence suggested that these strategies were effective in modifying risk.^22,23,24^ The real revolution, however, arrived with the 1994 report from the first of the statin megatrials, the Scandinavian Simvastatin Survival Study, which demonstrated that lowering low-density lipoprotein cholesterol (LDL-C) in secondary prevention reduced recurrent atherosclerotic event rates and ASCVD mortality.^25^ This ushered in the second wave of preventive cardiology. Subsequent randomized placebo-controlled statin trials demonstrated remarkably consistent results in essentially all patient groups. At this point, cholesterol management became central to the mission of ASCVD prevention.^26^ However, while evaluation and management of LDL-C remains a critically important aspect of CVD prevention, contemporary (ie, the third wave) preventive cardiology embraces the notion that LDL-C is only one component of a larger, more comprehensive evaluation and management strategy to mitigate ASCVD risk.

## Contemporary Preventive Cardiology

Until recently, prevention of ASCVD has centered around LDL-C prevention. Although this strategy has paid great dividends, it is well known that risk reduction with statins is incomplete. In fact, the recently-dubbed term “residual cardiovascular risk” refers to the fact that significant risk of cardiovascular events remains despite optimal statin therapy. The IMPROVE-IT trial (Improved Reduction of Outcomes: Vytorin Efficacy International Trial) examined whether the addition of ezetimibe to statin therapy could reduce rates of recurrent cardiovascular events in patients with recent acute coronary syndrome.^27^ Despite incrementally decreasing LDL-C levels by more than 20% and reducing cardiovascular event rates by 2% compared with the simvastatin monotherapy group, significant cumulative rates of recurrent cardiovascular events remained in the simvastatin-ezetimibe group after 7-year follow-up (32.7%).

**Figure 1:**
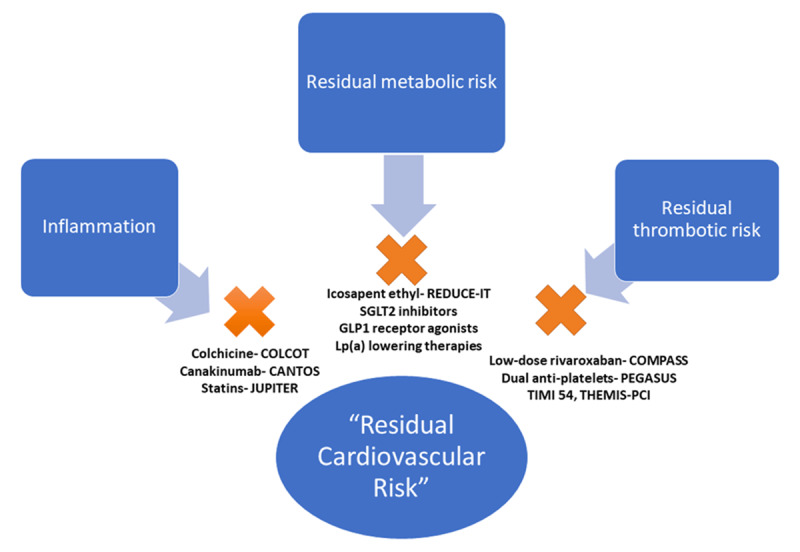
The pathways of residual cardiovascular risk, beyond traditional risk factors, with evidence-based therapeutic options. Reprinted with permission.^32^ COLCOT: Colchicine Cardiovascular Outcomes Trial; CANTOS: Canakinumab Anti-inflammatory Thrombosis Outcomes Study; JUPITER: Justification for the Use of Statins in Prevention: an Intervention Trial Evaluating Rosuvastatin; PEGASUS TIMI 54: Prevention of Cardiovascular Events in Patients with Prior Heart Attack Using Ticagrelor Compared to Placebo on a Background of Aspirin–Thrombolysis In Myocardial Infarction 54; THEMIS-PCI: The Effect of Ticagrelor on Health Outcomes in Diabetes Mellitus Patients Intervention Study-PCI; COMPASS: Cardiovascular Outcomes for People Using Anticoagulation Strategies; REDUCE-IT: Reduction of Cardiovascular Events With EPA–Intervention.

The cardiovascular outcome trials evaluating the proprotein convertase subtilisin/kexin type 9 (PCSK9) inhibitors offer the most poignant example of residual risk and the need to address all risk factors.^28,29,30^ In the FOURIER (Further Cardiovascular Outcomes Research with PCSK9 Inhibition in Subjects With Elevated Risk) trial, the PCSK9 inhibitor evolocumab was shown to incrementally improve cardiovascular outcomes in patients with stable ASCVD on background high-intensity statin therapy.^29^ However, despite achieving median LDL-C levels of 30 mg/dL with evolocumab, recurrent cardiovascular event rates (ie, residual risk) remained high (12.6% cumulative incidence of cardiovascular events at 3 years). Furthermore, a prespecified secondary analysis of the FOURIER trial demonstrated that while evolocumab could safely achieve single digit LDL-C concentrations, essentially eliminating LDL-C from the ASCVD risk equation, patients remained at relatively high risk.^31^ As such, while we are fortunate to have effective tools to dramatically lower LDL-C, the time has come to move beyond LDL-C and consider all of the categories of residual ASCVD risk (***Figure 1***).^32^ Fortunately, we find ourselves in a therapeutic renaissance that has the potential to dramatically attenuate ASCVD risk.

## Emerging Science in Cardiovascular Disease Prevention

In the past, the medical community had little to offer in terms of ASCVD risk assessment and management. Traditional quantitative risk assessment tools performed modestly, and management of ASCVD risk had been relatively narrow and nonspecific. We now have more precise methods to identify individuals who will most likely benefit from therapy and to tailor individualized therapeutic interventions. Significant advances have been made in subclinical atherosclerosis imaging, including coronary artery calcium scoring, coronary computed tomography angiography, and carotid artery ultrasound for intima-media thickness and plaque. Evaluation of multiple novel and promising biomarkers for ASCVD are underway.^33^ Genetic testing, whether through assessment of one or thousands of alterations in the genome, is gaining traction. In particular, there has been abundant interest in polygenic risk scores,^34,35^ which may be in clinical practice relatively soon. Additionally, there are a multitude of emerging therapeutics (***Table 1***)^36^ that address lipids, diabetes, weight, blood pressure, thrombosis, and inflammation by leveraging novel modalities such as monoclonal antibodies, antisense nucleotides, silencing ribonucleic acids, CRISPR-based techniques (clustered regularly interspaced short palindromic repeats), and vaccines. Moreover, the future of preventive cardiology should not be limited to prevention of atherosclerotic events but should expand to include prevention of heart failure and atrial fibrillation.^37,38^ Prevention of cognitive decline is another intriguing area of interest given the shared risk factor profile between dementia and ASCVD.

**Table 1:** Emerging targets and therapeutics to reduce ASCVD risk. Used and adapted with permission.^36^ ASCVD: atherosclerotic cardiovascular disease; GLP-1: glucagon-like peptide 1; LDL-C: low density lipoprotein-cholesterol; SGLT-2: sodium glucose cotransporter-2; siRNA: silencing ribonucleic acid; IL-1β: interleukin-1β.


COMORBIDITY	TARGET	THERAPEUTIC

Dyslipidemia	LDL-C, triglycerides, lipoprotein(a)	Bempedoic acid, monoclonal antibodies (alirocumab, evolocumab, evinacumab), siRNA (inclisiran), icosapent ethyl, antisense oligonucleotides (APOCIII-LRx, IONIS-APO(a)-LRx)

Inflammation	IL-1β	Canakinumab, colchicine

Diabetes	SGLT-2, GLP-1 receptor	Canagliflozin, dapagliflozin, empagliflozin, abiglutide, dulaglutide, exenatide, liraglutide, lixisenatide, semaglutide, tirzepatide

Obesity	Appetite, stomach capacity	Orlistat, locaserin, naltrexone-bupropion, phentermine-topiramate, liraglutide, tirzepatide, gastric bypass, sleeve gastrectomy, adjustable gastric band, biliopancreatic diversion with duodenal switch

Hypertension	Vasopeptidase, aldosterone synthase, soluble epoxide hydrolase, natriuretic peptide A, vasoactive intestinal peptide receptor 2, catheter-based interventions	Vaccines against angiotensin II and its receptor type I, renal denervation, baroreflex activation therapy

Thrombosis	Factor Xa, P2Y_12_ receptor	Rivaroxaban, ticagrelor


## Preventive Cardiology as a Dedicated Subspecialty

Given the burden, trends, and multidimensional nature of ASCVD, there has never been a greater need and opportunity for the creation of a dedicated subspecialty in cardiovascular disease prevention. Looking across the United States, one can observe that this is already happening spontaneously through new preventive cardiology and cardiometabolic medicine programs.^8,36,39^ However, while these secular trends are certainly a step in the right direction, preventive cardiology programs and subspecialty training remain fragmented and unstandardized. Moreover, preventive cardiology is not the sole domain of cardiologists; rather, programs greatly benefit from a broad range of specialists in endocrinology, internal medicine, preventive medicine, family medicine, and obstetrics/gynecology. Regardless of background, a preventive cardiologist of any stripe requires broad expertise in the core competencies of ASCVD risk assessment, lifestyle management, and ASCVD and comorbidity evaluation and treatment.^8,36^ ***Table 2*** provides a list of common referrals to a Center for Preventive Cardiology.

**Table 2:** Common referrals to a preventive cardiology center. ASCVD: atherosclerotic cardiovascular disease.


Personal or family history of ASCVD

ASCVD with recurrent events

Severe or difficult lipid disorders

Inherited lipid disorders (particularly children)

Genetic testing

Risk assessment with advanced lipid testing/novel biomarkers

Noninvasive atherosclerosis imaging to refine risk estimation

Statin-associated side effects

Lifestyle counseling (weight management, smoking cessation, diet, etc)

Healthy individuals interested in understanding and lowering ASCVD risk

Review and/or consultation for preventive medical therapies

Participation in clinical trials of novel preventive therapies


The optimal provision of cardiovascular disease prevention also requires direct integration of other healthcare team members including clinical pharmacists, registered dieticians, genetic counselors, and lifestyle coaches. While pharmacotherapy and other effective interventions are improving, greater emphasis and resources must be allocated to professional-grade lifestyle counseling. In addition, integrating clinical pharmacists into preventive cardiology practice increases both use and adherence to evidence-based preventive medications and markedly reduces patient costs.^40^ Furthermore, effective models such as the PCSK9 inhibitor clinic highlight the successful integration of clinical pharmacists in preventive cardiology with benefits to patients, medical practice, and healthcare systems.^41,42^ This model needs to be expanded well beyond the application of PCSK9 inhibitors to include the myriad effective, albeit expensive, drugs that are available to mitigate ASCVD risk.

Finally, the future of preventive cardiology as a dedicated subspecialty would greatly benefit by integrating the research emerging from the growing number of Centers for Preventive Cardiology. Interesting and/or perplexing cases should be enrolled in collaborative registries and potentially qualify for clinical trials. Prospectively establishing an integrated research network while the field of preventive cardiology is developing could ultimately lead to significant downstream improvements in ASCVD prevention.

## Conclusion

We have reached a critical juncture in ASCVD management where US mortality rates are reversing and improvements in intervention of manifest disease are yielding diminishing returns. Furthermore, the growing epidemics of obesity and diabetes stress the importance of a multidimensional approach to managing ASCVD and its risk factors. These disturbing trends call for a paradigm shift from intervention to prevention and have spontaneously led to current efforts to establish a dedicated subspecialty of preventive cardiology. Expertise in preventive cardiology goes well beyond evaluation and management of lipids and requires a distinct, specialized knowledge that spans multiple domains. Fortuitously, we find ourselves at the forefront of an exciting revolution in ASCVD prevention, with an unprecedented number of emerging innovations that vastly enhance ASCVD risk assessment and mitigation. We are just at the beginning, and the future is bright!

## Key Points

- Disturbing trends in cardiovascular risk factors (eg, rising rates of obesity and diabetes) and atherosclerotic cardiovascular disease (ASCVD) mortality (at a nadir and likely rising) in the United States coupled with diminishing returns in treating manifest disease serve as a call to action to focus on cardiovascular disease prevention.
- The medical community needs to shift its attention from intervention to multifactorial prevention.
- Preventing ASCVD requires multispecialty and multidisciplinary expertise, and this has led to recent efforts to develop the unique subspecialty of preventive cardiology.
- We are in the nascent stages of an exciting revolution in ASCVD prevention, with significant advances in risk assessment (eg, subclinical atherosclerosis imaging, novel biomarkers, genetic testing) and emerging targets and novel therapeutics (addressing lipids, diabetes, weight, blood pressure, thrombosis, and inflammation).
